# Human MiR-544a Modulates SELK Expression in Hepatocarcinoma Cell Lines

**DOI:** 10.1371/journal.pone.0156908

**Published:** 2016-06-08

**Authors:** Nicoletta Potenza, Filomena Castiello, Marta Panella, Giovanni Colonna, Gennaro Ciliberto, Aniello Russo, Susan Costantini

**Affiliations:** 1 Dipartimento di Scienze e Tecnologie Ambientali, Biologiche e Farmaceutiche, Seconda Università di Napoli, Caserta, Italia; 2 Servizio di Informatica Medica, Azienda Ospedaliera Universitaria, Seconda Università di Napoli, Napoli, Italia; 3 Direttore Scientifico, Istituto Nazionale Tumori “Fondazione G. Pascale”- IRCCS, Napoli, Italia; 4 CROM, Istituto Nazionale Tumori “Fondazione G. Pascale”—IRCCS, Napoli, Italia; National Institutes of Health, UNITED STATES

## Abstract

Hepatocellular carcinoma (HCC) is a multi-factorial cancer with a very poor prognosis; therefore, there are several investigations aimed at the comprehension of the molecular mechanisms leading to development and progression of HCC and at the definition of new therapeutic strategies. We have recently evaluated the expression of selenoproteins in HCC cell lines in comparison with normal hepatocytes. Recent results have shown that some of them are down- and others up-regulated, including the selenoprotein K (SELK), whose expression was also induced by sodium selenite treatment on cells. However, so far very few studies have been dedicated to a possible effect of microRNAs on the expression of selenoproteins and their implication in HCC. In this study, the analysis of SELK 3’UTR by bioinformatics tools led to the identification of eight sites potentially targeted by human microRNAs. They were then subjected to a validation test based on luciferase reporter constructs transfected in HCC cell lines. In this functional screening, miR-544a was able to interact with SELK 3’UTR suppressing the reporter activity. Transfection of a miR-544a mimic or inhibitor was then shown to decrease or increase, respectively, the translation of the endogenous SELK mRNA. Intriguingly, miR-544a expression was found to be modulated by selenium treatment, suggesting a possible role in SELK induction by selenium.

## Introduction

Hepatocellular carcinoma (HCC) is the third leading cause of cancer deaths worldwide [1]. It is a multi-factorial cancer and its initiation can be attributed to a wide range of factors among which hepatitis B and C virus, cirrhosis, aflatoxin and type 2 diabetes [2]. Even if in the last years some progresses were reached about the understanding of the molecular mechanisms at the basis of HCC development and, hence, there were improvements in its therapy, the prognosis remains poor due to a high recurrence. Certainly, an improved understanding of its pathogenesis would facilitate the development of more effective outcomes for HCC diagnosis and treatment at early stages [3]. In fact, some Authors are searching to identify new markers, and, recently, also the hepatic C1B receptor (CB1R) was found up-expressed in HCC patients and its blockade was suggested as a potential therapeutic target [4]. Many studies have shown an important role of microRNAs (miRNA) in HCC [5–8].

miRNAs are small, interfering, and non-coding RNA of length equal to 21–30 nucleotides, and approximately 1000 of these sequences have been found in the human genome [9]. They regulate negatively the gene expression by binding to complementary mRNAs, thus affecting their stability and translation [10]. Several studies have demonstrated that miRNAs are involved in human cancer, such as lung, breast, brain, liver, colorectal cancer, and leukemia [5, 11–15]. By targeting different genes in tumor development, miRNAs can function as either oncogenes or tumor suppressor [16–17].

A recent review has reported a summary about the role of miRNAs detected in liver carcinogenesis, and in HCC diagnosis, therapy and prognosis. Among these there are thirteen miRNAs identified as oncomiRs and, hence, able to induce tumor initiation by down-regulation of its suppressor genes, and ten miRNAs named anti-oncomiRs, which target oncogenes and block tumor invasion[18].

Recently, our group focused its attention on the selenoprotein family and their involvement in HCC [19], considering that selenium is able to modulate the oxidative stress, known to induce firstly cell damage and, then, cancer. In particular, 25 selenoproteins were found in humans, and the most of them is involved in detoxification, redox regulation, viral suppression, immune-system protection and even cancer [20–21]. Recently, we evaluated the seleno-transcriptome expression in two human HCC cell lines, HepG2 and HuH-7, commonly used as model of liver cancer in absence of viral infection. Our data showed that in both cell lines there are three down-regulated and ten up-regulated genes, among which also the selenoprotein K (SELK) [19]. This small protein of the endoplasmic reticulum (ER) is involved in the cellular redox state control and signaling as well in the control of oxidative stress, ER calcium flux through endoplasmic reticulum, and ER-associated protein degradation (ERAD) pathway [22]. Moreover, SELK seems also able to catalyze protein palmitoylation reactions [22]. In the ERAD system it is, although not yet entirely clear, operatively associated with p97(VCP) and SELS (Selenoprotein S), in the organization of the supra-molecular ERAD complex, and, in particular, when associated to p97(VCP), it has been found involved in moving out the misfolded proteins from the ER to the cytosol [23].

Although no detailed information is present in the literature about its involvement in cancer, some studies have suggested a correlation with the tumor progression, and, in particular, its polymorphisms were associated with the prostate cancer risk [24]. In particular there are only two studies about the SELK involvement in HCC by using HCC cell lines [25–26]. In details, some Authors showed that in HepG2 cell line SELK was regulated by two ER stressing agents, tunicamycin and β-mercapto-ethanol, and its silencing was able to increase cell death and apoptosis [25]. Then, our group demonstrated that SELK expression is correlated with the increase of the sodium selenite concentration in two HCC cell lines, HepG2 and HuH-7 [26].

However, no information are reported about miRNAs targeting the mRNAs of selenoproteins, including SELK. Therefore, we have performed a prediction for the target sites of human miRNAs within 3’UTR of SELK by means of bioinformatic tools. Then, these sites were verified by a validation test based on luciferase reporter constructs transfected in HCC cell lines. We decided to start with a study on HCC cell lines to perform a target validation even because the etiology of this cancer is very complex and the enrollment and clinical characterization of HCC patients require a long time. This screening of target validation revealed that miR-544a was able to silence the SELK expression in HCC cell lines. Hence, we focused on miR-544a, demonstrating that it interferes with SELK translation and is modulated by selenium treatment.

## Materials and Methods

### Reporter constructs

The SELK 3’UTR segments that were supposed to be targeted by human miRNAs were obtained by chemical synthesis of complementary oligonucleotides (Invitrogen) containing upstream *Xho*I and *Eco*RV restriction sites and a downstream *Not*I site [27]. Each couple of oligonucleotides, representing the target sites for the different miRNAs reported in Table 1, was annealed, 5’-phosphorylated with T4 polynucleotide kinase (Roche), and ligated into *Xho*I and *Not*I sites of psiCheck-2 (Promega). Digestion with *Eco*RV were then used to screen the recombinant clones; selected constructs were sequenced to confirm their identity. Control plasmid (indicated as I) for miR-544a was obtained by the same approach, i.e. cloning the couple of oligonucleotides representing the inverted target sequence (5’-TAAGACGTTGGATTTTTATAAA-3’) in psiCheck-2 vector.

**Table 1:** Human microRNAs with potential binding sites on SELK 3’UTR.

Human miRNA	SELK 3'UTR position*	MiRNA-mRNA pairing**
**miR-632**	6–24	3' AGGGUGUCCUU**CGUCUGU**G 5'
		5' UGCUCUAAGAAGCAGACAA 3'
**miR-181a-5p**	78–101	3' UGAGUGGCUGUCG-CA**ACUUACA**A 5'
		5' CAUGAUGAGCAGUAGAUGAAUGUG 3'
**miR-181b-5p**	75–101	3' UGGGUG—GCU-GUCGU-U**ACUUACA**A 5'
		5' GACCAUGAUGAGCAGUAGAUGAAUGUG 3'
**miR-338-3p**	135–156	3' GUUGUUUUAGUGAC**UACGACC**U 5'
		5' AGAUGAAUGAGGUCAUGCUGGG 3'
**miR-381-3p**	212–235	3' UGUCUCUCGAACGG—**GAACAUA**U 5'
		5' UUUGUCUCAUUACCUUUUUGUAUA 3'
**miR-200b-3p**	230–251	3' AGUAGUAAUGGUCCG**UCAUAA**U 5'
		5' UGUAUAGUUUAUUAAAGUAUUA 3'
**miR-544a**	269–290	3' CUUGAACGAUUUUU**ACGUCUU**A 5'
		5' AAAUAUUUUUAGGUUGCAGAAU 3'
**miR-340-5p**	290–309	3' UUAGUCAGAGUAAC**GAAAUAU**U 5'
** **		5' UGGACUCCUCAU—CUUUAUAU 3'

*Nucleotide numbering starts after stop codon.

**The seed region of miRNAs is marked in bold.

### Cell cultures and transfections

Human hepatocarcinoma cell lines, HepG2 and HuH-7, were cultured in RPMI 1640 and DMEM respectively, containing 10% fetal bovine serum, 2mM L-glutamine, 50 U/ml penicillin and 100 mg/ml streptomycin; PLC/PRF/5 cells were cultured as HuH-7 with the addition of non-essential amino acids (0.1 mM) in the medium. The day before transfection, the cells were trypsinized and seeded in medium without antibiotics in 12-well plates.

Transfections were performed with cells at 80–90% of confluence by using 3 μl of Lipofectamine 2000 (Invitrogen) for 1 μg of nucleic acids, as described by the manufacturer. HepG2 and HuH-7 cells were transfected with 0.2 and 0.05 μg of reporter constructs, respectively; miScript miR-544a mimic, miScript miR-544a inhibitor, and their controls with unrelated sequences (AllStars Negative Control and miScript Inhibitor Negative Control, respectively; all from Qiagen) were transfected at 50nM along with 50ng of phRL-tk plasmid encoding *Renilla luciferase* to monitor transfection efficiency. After 6 h, transfection mix was replaced with complete medium. The analyses were performed 48h after transfection.

### RNA purification, real-time PCR analyses and luciferase assays

Total RNA was extracted by miRNeasy mini kit (Qiagen) from cell cultures according to the manufacturer’s protocol.

MiRNAs were quantified along with RNU6B (reference gene) by RT-qPCR with TaqMan® miRNA assays from Applied Biosystems according to the manufacturer’s protocol. Their expression level were normalized to that of the reference gene by using the 2^-ΔCt^ method.

Luciferase assays were performed using the Dual-Luciferase Reporter Assay System (Promega) according to the manufacturer’s protocol.

All the analyses (qPCR and luciferase assays) were performed on three independent experiments, each in triplicate.

Comparison of the data sets was performed by Student’s *t*-test and a value of p<0.05 was considered significant.

### Western blot analysis

Total proteins from cultured cells were prepared in lysis buffer (20 mM Tris–Cl, pH 7.5, 150 mM NaCl, 1 mM EDTA and NP40) containing a protease inhibitor cocktail (Roche). 40 μg proteins were separated by 15% SDS–polyacrylamide gel electrophoresis and transferred to nitrocellulose membrane (GE Healthcare) following standard protocols. Antibody against SELK (Abcam) and γ-tubulin (Sigma–Aldrich) were used for immunodetection according to the manufacturer’s instructions. Protein bands were visualized by incubating the blots with the horseradish peroxidase conjugated anti-rabbit antibody (Santa Cruz Biotechnology) and by using Immobilon™ Western Chemiluminescent HRP Substrate (Millipore) according to the manufacturer’s instructions. Blot images were captured by ChemiDoc XRS (Bio-Rad) and protein bands were quantified by Image Lab software (Bio-Rad).

## Results

### Searching for target sites of human microRNAs within 3’UTR of SELK

On the basis of the SELK up-expression in HCC cell lines when compared to normal hepatocytes [19] and to assess a possible regulation of SELK by miRNAs, we have first performed a prediction of target sites for human miRNAs within 3’UTR of SELK, and, then, we tested the sequence targets for responsiveness to hepatic miRNAs.

The computational analysis was performed by two tools, miRanda [28] and Targetscan [29], by scanning the 3’UTR of SELK for the presence of target sites within human miRNAs. We restricted the results (i) to the targets predicted by both programs, (ii) to miRNA-mRNA pairings with a perfect matching of the seed region (2-8nt of miRNA), and (iii) to miRNAs expressed in the liver or in cultured hepatic cell lines. The analyses yielded eight top-ranking target sites (**Table 1**).

These target sites were subjected to a validation test, based on luciferase reporter constructs transfected in the HCC cell lines. In details, 3’ UTR segments, containing putative miRNA target sites, were separately cloned downstream of the *Renilla reniformis* luciferase (Rl) coding sequence, contained in the psi-Check-2 vector. One reporter construct was prepared for both miR-181a-5p and miR-181b-5p, since their target sites resulted overlapped. The recombinant plasmids were then transfected in HuH-7 cells and the luciferase activity was measured 48h after the transfection. We have decided to transfect the HuH-7 cell line, because it is a cell line very used as experimental HCC model as well as very similar to the cancerous tissues of HCC patients; in detail, it is originated from a liver tumor of a 57 year old Japanese male, and its cells are well differentiated. Recent studies have also shown that this cell line is associated with a low expression of cytokeratin 8/18 (CK8/18) when compared to normal hepatocytes [30]; moreover, it shows also a p53 with a constitutive mutation A:T->G:C at codon 220 that characterizes a strongly malignant phenotype [30].

The rationale for using the luciferase-based assay is that the binding of a given hepatic miRNA to the SELK target, when transcribed together with the luciferase coding sequence, will repress the production of the reporter protein, thus reducing the luciferase activity in respect to the control. In these experiments, the control was the psiCheck-2 vector that did not contain any SELK sequence. In addition, the *Renilla* luciferase signal was normalized to that of *Photinus pyralis* luciferase (Luc), whose gene is also contained in the psiCheck-2 vector and used as an intra-plasmid transfection normalization reporter.

**Fig 1:**
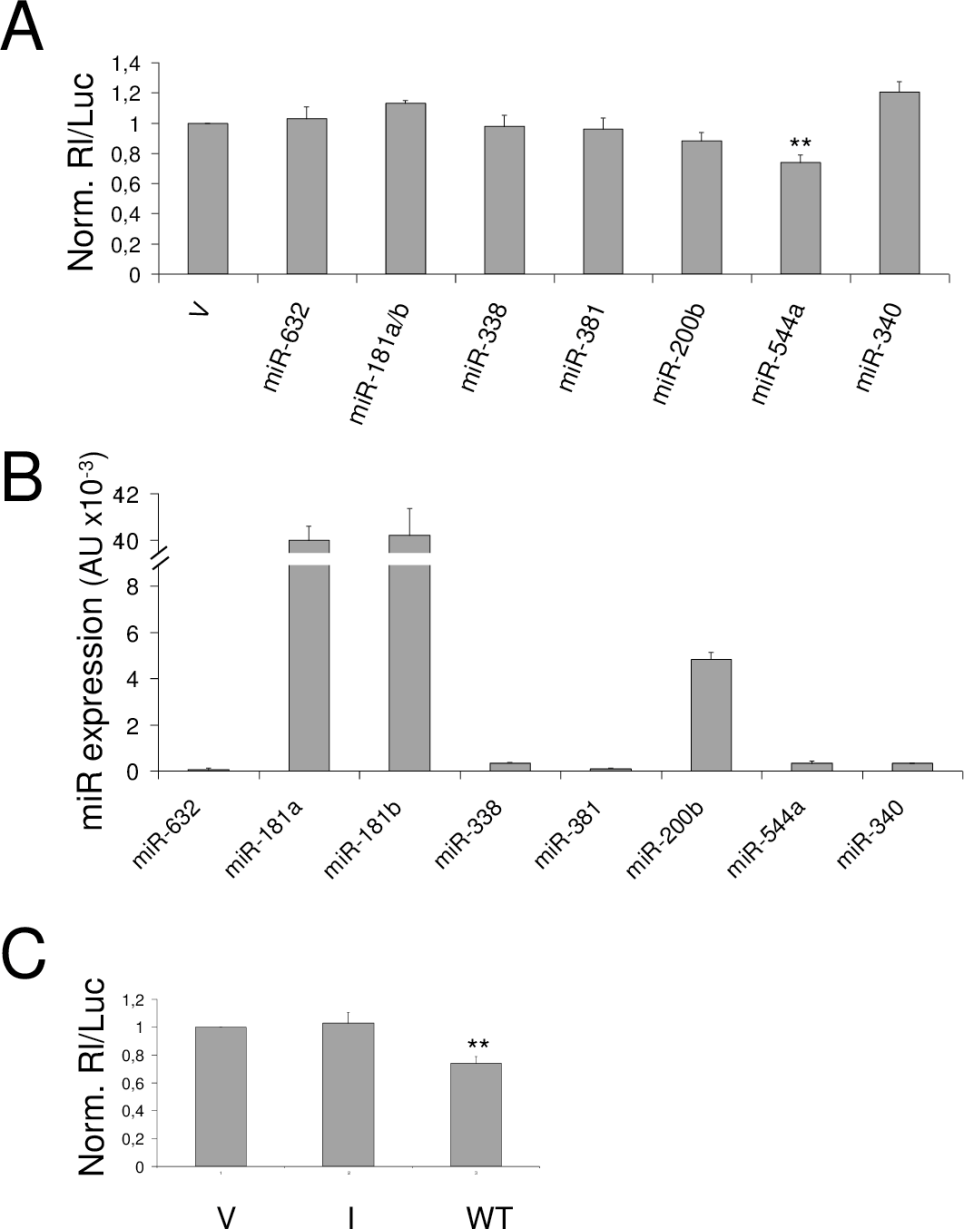
Testing of 3’ UTR SELK targets for responsiveness to human hepatic miRNAs in HuH-7 cell line. **(A)** The potential targets of indicated miRNAs were cloned in the reporter vector psiCheck2. One construct was used for both miR-181a and miR-181b (miR-181a/b), since their target sequences resulted overlapped. 48h after transfection, luciferase activities were recorded; the *Renilla* luciferase activity (Rl) was normalized to the firefly luciferase activity (luc) and the uninhibited activity relative to the parental vector psiCheck2 (V) was set to 1. (B) Expression of miRNAs were evaluated by qRT-PCR and reported as arbitrary units (A.U.) **(C)** Following the same procedure as above (A), a control construct (I), containing the inverted sequence targeted by miR-544a, was tested. P-values < 0.01 were evaluated by Student’s t-test and indicated with **.

Under these experimental conditions, the 3’ UTR SELK segment 269–290, which is supposed to be targeted by miR-544a, inhibited significantly the expression of the reporter (Fig 1A); in particular, the activity registered for the reporter construct was found 26% lower than that obtained with the parental vector, indicating a direct interaction between miR-544a and the SELK sequence.

To verify whether the silencing effect was merely due to difference in the expression level of the miRNAs targeting the different reporter constructs, we also analyzed their expression by RT-qPCR (Fig 1B). miR-632, miR-338, miR-381 and miR-340 expression resulted very low, suggesting an alternative explanation to the absence of silencing effect on their predicted targets, as for example we can hypothesize that they do not work at physiological concentration of the HCC cell context. In contrast, miR-181a and miR-181b had higher expression level than miR-544a and target the same reporter construct; however, no silencing effect was observed. Hence, we can highlight that the silencing effect observed for miR-544a construct depend on its ability to interact with SELK sequence and was strong, taking into account the relative expression levels of the different miRNAs.

As a further control, the reporter activity was also registered after transfection of a construct containing the inverted sequence recognized by miR-544a (I); the luciferase activity was found to be comparable to that determined after transfection of the parental vector and significantly higher than that obtained with the wild-type construct (WT) (Fig 1C), as expected. The I construct was then used as a control for the next experiments, allowing to register the maximum value of the uninhibited reporter activity.

Overall, the screening of the SELK target sequences indicates that at physiological concentration miR-544a is able to silence the reporter gene by interacting with the 3’ UTR SELK segment 269–290.

### Expression of miR-544a in HCC cell lines

**Fig 2:**
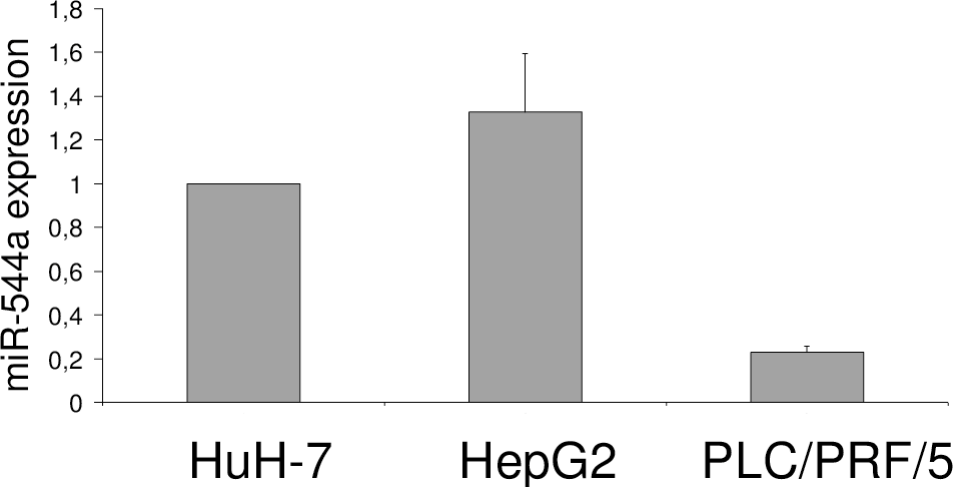
miR-544a expression in three common HCC cell lines. miR-544a expression levels were evaluated by RT-qPCR and reported as fold change relative to that of HuH-7.

Since no literature data are known about miR-544a involvement in HCC, we evaluated also its expression levels in some common HCC cell lines. In details, having investigated the silencing effect of miR-544a in HuH-7 cells, we compared the expression of this miRNA with other two HCC cell lines showing a different phenotype with respect to HuH-7 cells with no viral infection but p53 mutation (mutant p53 Y220C) [30]. In particular, the analyses were performed on HepG2 cell line with no viral infection but carrying wild-type p53 and with a CK8/18 expression similar to that of normal hepatocytes [30], and on the PLC/PRF/5 cell line, containing several copies of HBV DNA integrated in its genome [31] and carrying a mutated p53 gene (mutant p53 R249S) [32]. As shown in Fig 2, miR-544a shows a comparable level of expression in HuH-7 and HepG2 cells, and a lower extent in PLC/PRF/5. This confirms that the etiology of HCC is complex as well the need of studying all the possible phenotypes. In this study we focused on cell lines without viral infection because in the last years there are increasing the patients that develop HCC without viral origin as well as the necessity to identify new targets to develop new potential therapeutic agents [33].

### Silencing activity of hsa-miR-544a

**Fig 3:**
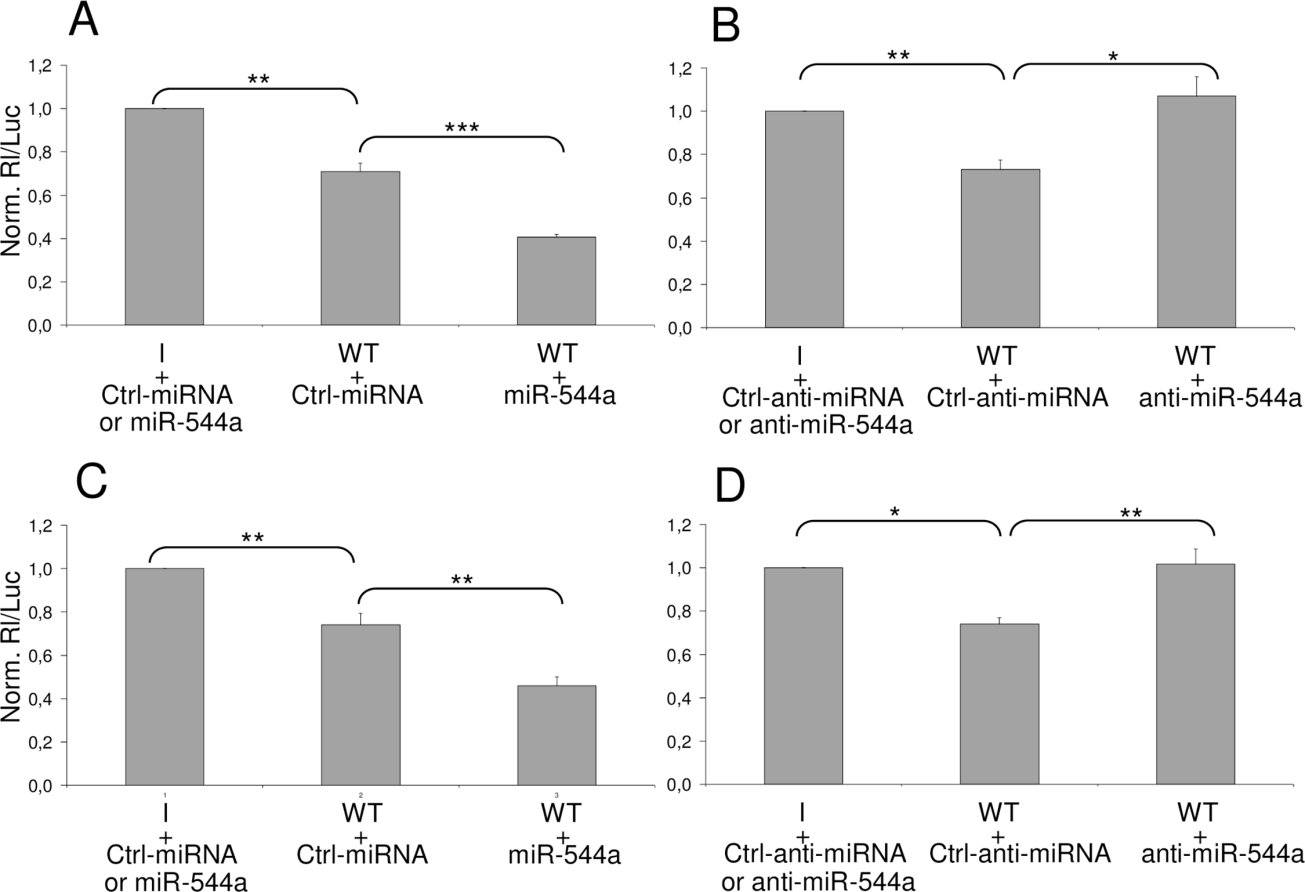
Interfering activity of miR-544a in HCC cell lines. HuH-7 and HepG2 cells were transfected with the luciferase-based reporter plasmid psiCheck-2 containing the SELK target sequence for miR-544a (WT) or a control DNA with inverted sequence (I), along with 50 nM miR-544a mimic (miR-544a, A and B) or 50 nM miR-544a inhibitor (anti-miR-544a, C and D). Ctrl-miRNA and Ctrl-anti-miRNA are their relative unrelated molecules used as negative controls. Significant p-values evaluated by Student’s t-test were indicated with *, ** and *** when P < 0.05, or < 0.01 or < 0.001.

The silencing effect of miR-544a was then investigated by transfecting both reporter constructs (I and WT) along with miR-544a mimic and miR-544a inhibitor, as well as their negative control molecules, Ctrl-miRNA and Ctrl-anti-miRNA, which do not match the target sequence. MiR-544a mimic increased the level of endogenous miR-544a of approximately 300-fold, as determined by RT-QPCR (*data not shown*). This boost resulted in an additional luciferase reduction on the reporter construct WT (about 30%) in comparison to the inhibition due to the endogenously expressed miR-544a (see the results obtained by transfecting WT construct together with the negative control mimc, Ctrl-miRNA), leading to a total inhibition equal to 60% (Fig 3A). Conversely, the inhibition of miR-544a activity resulted in a recovery of luciferase activity when it was compared to that measured by transfecting the WT construct together with the negative control inhibitor (Ctrl-anti-miRNA) (Fig 3B).

These experiments were also performed in HepG2 cell line (Fig 3C and 3D) to generalize the results, also considering the comparable expression levels of miR-544a in HuH-7 and HepG2 cells (Fig 2), and to verify that this miRNA is not influenced from wild-type or mutated p53. The data clearly indicate that the silencing effect of cellular miRNAs toward the 3’ UTR SELK segment 269–290 can be attributed to miR-544a in both tested HCC cell lines.

**Fig 4:**
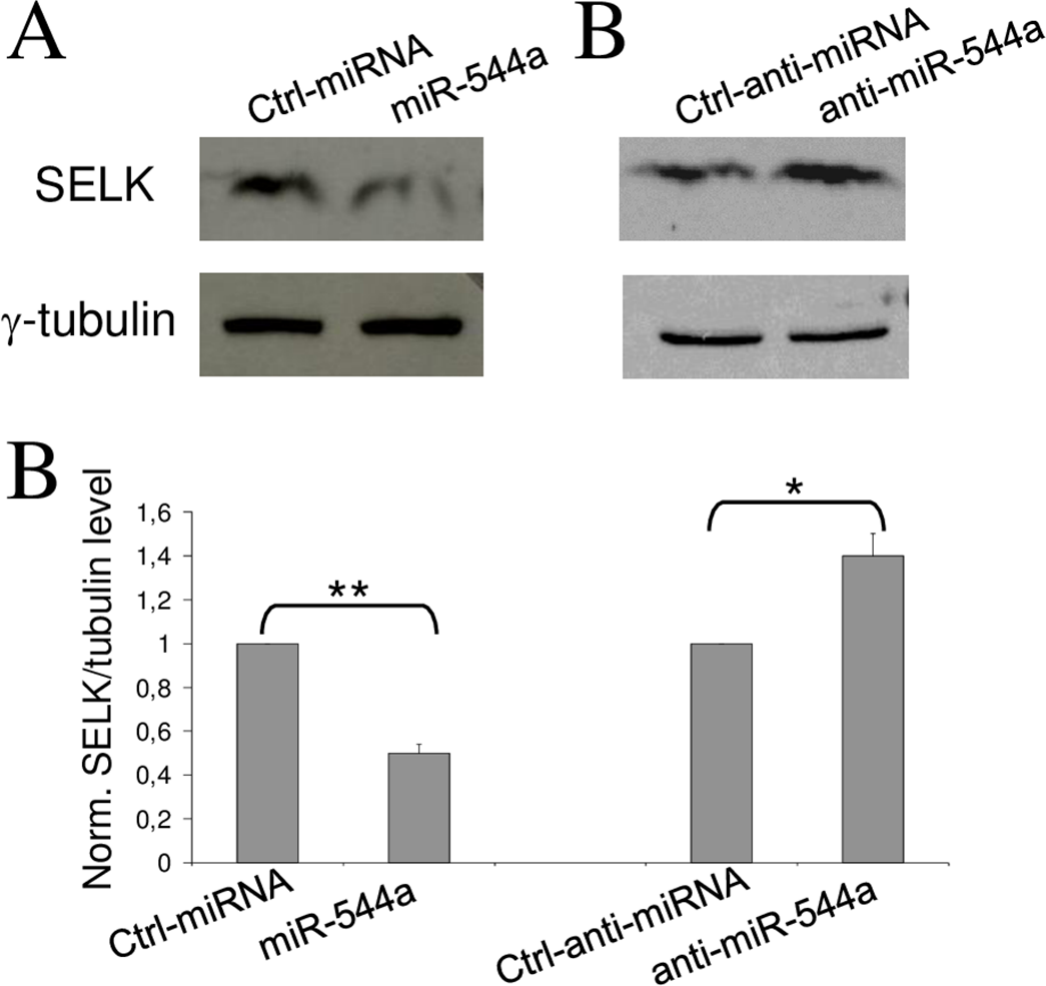
The silencing effect of miR-544a on SELK in HuH-7 cells. Representative Western blot analysis of protein extracts at 48 h after transfection with miR-544a mimic (A) or miR-544a inhibitor (B) and their relative control molecules at 50 nM. (C) Quantification of SELK protein band normalized to that of tubulin; signal intensity values determined for the control experiments (Ctrl-miR and Ctrl-anti-miR) were set at 1; values represent the mean ± s.d. of three independent experiment. P-values are indicated as described in Fig 3.

The assays described above are based on the silencing effect of miR-544a on the translation of a chimeric mRNA containing the *Renilla* luciferase coding sequence and a segment of SELK UTR mRNA. However, it was not known whether the same miRNA could be able to interfere with the translation of the natural mRNA. This point was addressed by transfecting cells with miR-544a mimic, or inhibitor, and evaluating their effect on the SELK protein. Western blot analyses revealed a reduced amount of SELK as a consequence of miR-544a mimic transfection and an increase of SELK after miR-544a inhibitor transfection (Fig 4). These results clearly indicate that miR-544a is able to interfere with the SELK translation providing direct evidence that the interaction between the selected miRNA and SELK occurs naturally in hepatic cells.

A possible effect of miR-544a mimic and inhibitor on cell proliferation was evaluated by MTT assay. No difference was observed on the proliferation of HuH-7 and HepG2 cell lines transfected with miR-544a mimic and inhibitor compared to their relative control molecules, as already reported for other cell lines (see S1 Fig) [34].

### Effect of sodium selenite on miR-544a interfering activity

Selenium is present in the selenoproteins as selenocysteine, but many aspects of the selenoprotein biosynthesis remain to be elucidated. Further, little is known about a potential regulation of selenoproteins by miRNAs. In particular, only for human colon adenocarcinoma cells (Caco-2) the expression of a panel of miRNAs and selenoproteins has been evaluated after selenium treatment [34]. These same Authors have also shown that selenium intake could alter the expression of some miRNAs, where miR-185 likely plays a role in the regulation of GPX2 and SEPHS2 expression [35].

**Fig 5:**
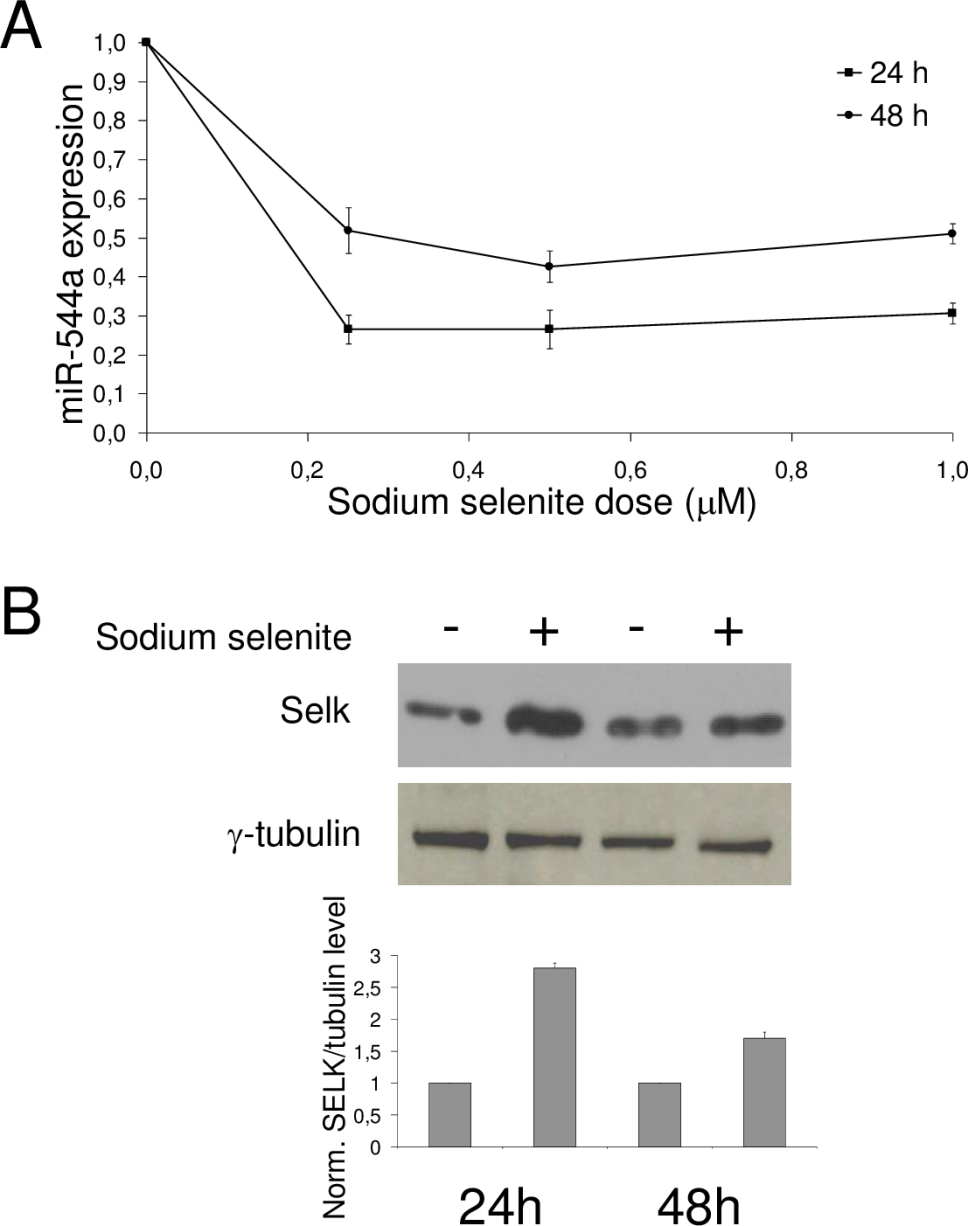
**Effect of sodium selenite on miR-544a expression and SELK** (A) Cells were cultured for 24 h and 48 h in standard condition or in presence of increasing sodium selenite doses; miR-544a expression was determined by RT-qPCR and reported as fold change of expression in treated samples vs untreated ones. (B) Upper panel, representative Western blot analysis of protein extracts from cells cultured in absence (-) or presence (+) of 1 μM sodium selenite for 24h and 48 h; lower panel, quantification of protein bands; values represent the mean ± s.d. of three independent experiment.

Recently, we have shown that different amounts of sodium selenite treatment in HepG2 and HuH-7 cell lines produced a rapid increase in the SELK level after 24 h [26]. We investigated whether miR-544a expression was modulated by selenium and what was the selenium role in the induction of SELK. In details, we treated HuH-7 cells with concentrations of 0.25, 0.5 and 1 μM of sodium selenite, as already used [26], incubating for 24 h and 48 h. The concentrations of sodium selenite were chosen on the basis that human physiological concentration is less than 3 μM [26]. Real-time PCR analyses revealed that miR-544a was sensitive to the selenium (Fig 5A). In particular, 24 h after treatment, even at 0.25 μM sodium selenite exerts a repressive effect on the expression of miR-544a (approximately 3.5-fold inhibition), when compared to untreated samples. The repressive effect was also observed 48h after treatment. Conversely, SELK levels increased after 24h and 48h of treatment (Fig 5B). Overall, these results suggest that miR-544a could be in part responsible of SELK induction by sodium selenite.

## Discussion

Selenoproteins are emerging as an important protein family involved in detoxification, redox regulation, viral suppression, immune-system protection, in healthy humans as well as in cancer [20–21]; however, their biological functions remain largely to be investigated. Although it is known that the HCC pathogenesis clearly involves miRNA activities [5–8], no data have been still reported on the possible regulation of selenoproteins by miRNAs. The present study is focused on the search for miRNAs able to target SELK-mRNAs. Our experimental approach started from bioinformatics predictions about potential target sites of hepatic miRNAs in the 3’UTR of SELK. Then, we have performed a functional screening based on the target validation in HCC cell lines. Overall, our data evidenced that: i) only miR-544a was able to silence, at physiological concentration, the reporter gene by interacting with the 3’ UTR SELK segment 269–290 (Fig 1A), ii) the observed silencing effect was specifically due to miR-544a, because it was modulated by miR-544a mimic and inhibitor (Fig 1B), iii) miR-544a is able to interfere with SELK translation through a naturally occurring interaction in hepatic cells (Figs 3 and 4).

Furthermore, since SELK was found to be induced by sodium selenite [26], we have also investigated whether miR-544a was responsive to selenium, finding that both miR-544a and SELK expression were conversely regulated by sodium selenite. In particular, it is very important to underline that, as shown in Fig 5, after the treatment of HCC cell lines with sodium selenite, the gene expression trend of SELK was towards increasing, while those of miR-544a towards a decrease, thus suggesting that the selenium is able to block the ability of miR-544a to negatively regulate the SELK expression.

This result appears very interesting considering that HCC is still a fatal disease because no adequate treatment is available and many studies are aimed to identify new tumor markers and new therapeutic targets without adequate actions on the knowledge of fundamental biological mechanisms. An example is given by the recent transcriptomic analysis performed on HCC tissues in comparison with normal controls that evidenced the up-expression of hepatic levels of CB1 receptors, thus suggesting their crucial role in the induction of some tumor-promoting genes as well as that their genetic ablation could have a therapeutic potential [4]. In addition some pre-clinical and clinical studies are involved into the evaluation of the role of miRNAs in HCC carcinogenesis and their utility as therapeutic targets or diagnostic and prognostic markers [18]. For this reason, some Authors reviewed the set of miRNAs known to be involved in HCC initiation and progression defining them as hepato-miRnoma [18]. They have also reported the list of thirteen miRNAs, up-expressed in HCC tissues and sera, naming them as hepato-onco-miRs, due to their ability to induce HCC development by blocking the expression of tumor suppressor genes. Moreover, other ten miRNAs, mainly down-expressed in HCC tissues and sera, were similarly defined as hepato-anti-onco-miRs, because they target oncogenes and inhibit the cancer growth [18]. On the other hand, it is still unknown how these miRNAs regulate the tumor pathways. Recently, it has been studied the role of miRNAs in the p53 network, demonstrating that 33 of them are significantly regulated by p53 in HepG2 cell line, when treated with the doxorubicin, thus providing new insights about the p53-miRNA regulatory role in the HCC network [36].

However, in this context it is worth of note to underline that in all these studies, the miR-544a was not reported, and, hence, our results indicates for the first time its involvement in HCC. In general, the biological function(s) of miR-544a is still largely unknown, since scarce data are reported in the literature. In particular, Mo et al. (2014) [37] reported that miR-544a down-regulates CDH1 (E-cadherin) and up-regulates vimentin in non-small cell lung cancer (NSCLC). Moreover, this miRNA was also found able to reduce the GSK3β expression, always in lung cancer stem cells [38], as well as of activating the Wnt signaling pathway in gastric cancer cells through the induction of epithelial-mesenchymal transition (EMT) and the expression of VIM, SNAI1 and ZEB1 [34]. The(se) same Authors have also shown that miR-544a, while decreasing the CDH1 and AXIN2 expression, at the same time induces the nuclear import of β-catenin and stabilizes β-catenin in the nucleus by means of Wnt signaling pathway activation [34]. Furthermore, the role of miR-544a was tested also in a hypoxic breast cancer model because it was able to silence the mammalian target of rapamycin (mTOR) [39]. Finally, this miRNA has been found down-expressed in cervical cancer tissues compared with normal cervical tissues, and directly targeting the YWHAZ oncogene, hence developing its function as a tumor suppressor [40].

In conclusion, we suggest for the first time that a miRNA is able to interfere with SELK translation in HCC cell lines. Of course, we are well aware that further studies are needed to measure the abundance of SELK and miR-544 in HCC samples from patients with diverse etiology as well as at the various stages of the cancer to unravel the possible role of miR-544a in signaling pathways specific for HCC. In particular, these studies could also highlight whether miR-544a and/or SELK expression level might be useful for improving the diagnosis and/or prognosis of this cancer.

Moreover, on the basis of data related to the treatment of HCC cells with sodium selenite, we can suggest an intriguing network, among between selenium, as essential micronutrient, a miRNA, i.e., miR-544a, and a protein, i.e., SELK. In fact, selenium intake, being capable to alter the expression of a miRNA, should interfere with the expression of target genes, in this case with SELK. This may allow to evidence how epigenetic mechanisms, miRNA-mediated, could have a role in metabolic regulation exerted by micronutrients.

## Supporting Information

S1 Fig Effect on cell proliferation of miR-544a. HuH-7 cells were transfected with miR-544a mimic or anti-miR-544a and their relative control molecules at 50 nM. After 48 hours, cell growth was estimated by MTT assay.
(DOCX)
