# Supplementary material for: Human MiR-544a Modulates SELK Expression in Hepatocarcinoma Cell Lines
Source: PLoS One. 2016 Jun 8;11(6):e0156908. doi: 10.1371/journal.pone.0156908 (PMC4898719; doi:10.1371/journal.pone.0156908)
Supplement: S1 Fig — HuH-7 cells were transfected with miR-544a mimic or anti-miR-544a and their relative control molecules at 50 nM. After 48 hours, cell growth was estimated by MTT assay. (DOCX) [file pone.0156908.s001.docx]

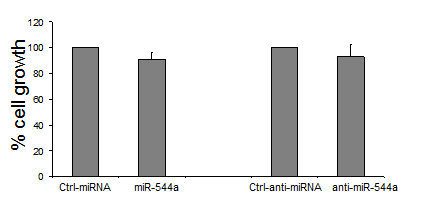


**Fig. S1. Effect on cell proliferation of miR-544a.** HuH-7 cells were transfected with miR-544a mimic or anti-miR-544a and their relative control molecules at 50 nM. After 48 hours, cell growth was estimated by MTT assay. In details, the cells were plated in 96-well microtiter plates and their growth was evaluated by quadruplicate analysis with MTT assay, as detailed below. 50 µL of 1mg/ml 3-(4,5-Dimethylthiazol-2-yl)-2,5-Diphenyltetrazolium bromide (MTT) were mixed with 200 μL of medium and added to the well. After 4 hours of incubation at 37 °C, the medium was removed and the purple formazan crystals produced in the viable cells were solubilized in 100 μL of dimethyl sulfoxide and quantitated by measurement of absorbance at 570 nm with a plate reader.
